# Measurement Invariance of the WHODAS 2.0 in a Population-Based Sample of Youth

**DOI:** 10.1371/journal.pone.0142385

**Published:** 2015-11-13

**Authors:** Melissa Kimber, Jürgen Rehm, Mark A. Ferro

**Affiliations:** 1 Department of Clinical Epidemiology and Biostatistics, McMaster University, Hamilton, Ontario, Canada; 2 Social and Epidemiological Research Department, Centre for Addiction and Mental Health, Toronto, Canada; 3 Dalla Lana School of Public Health, University of Toronto, Toronto, Canada; 4 PAHO/WHO Collaborating Centre for Mental Health & Addiction, Toronto, Ontario, Canada; 5 Klinische Psychologie and Psychotherapie, Technische Universität Dresden, Dresden, Germany; 6 Department of Psychiatry and Behavioural Neurosciences, McMaster University, Hamilton, Ontario, Canada; 7 Department of Pediatrics, McMaster University, Hamilton, Ontario, Canada; University of Perugia, ITALY

## Abstract

The World Health Organization Disability Assessment Schedule 2.0 (WHODAS 2.0) is a brief measure of global disability originally developed for adults, which has since been implemented among samples of children and youth. However, evidence of its validity for use among youth, particularly measurement invariance, is lacking. Investigations of measurement invariance assess the extent to which the psychometric properties of observed items in a measure are generalizable across samples. Satisfying the assumption of measurement invariance is critical for any inferences about between-group differences. The objective of this paper was to empirically assess the measurement invariance of the 12-item interview version of the WHODAS 2.0 measure in an epidemiological sample of youth (15 to 17 years) and adults (≥ 18 years) in Canada. Multiple-group confirmatory factor analysis using a categorical variable framework allowed for the sequential testing of increasingly restrictive models to evaluate measurement invariance of the WHODAS 2.0 between adults and youth. Findings provided evidence for full measurement invariance of the WHODAS 2.0 in youth aged 15 to 17 years. The final model fit the data well: χ^2^(159) = 769.04, *p* < .001; CFI = 0.950, TLI = 0.958, RMSEA (90% CI) = 0.055 [0.051, 0.059]. Results from this study build on previous work supporting the validity of the WHODAS 2.0. Findings indicate that the WHODAS 2.0 is valid for making substantive comparisons of disability among youth as young as 15 years of age.

## Introduction

More than ever, youth are being diagnosed and living with chronic health conditions and physical disabilities [1]. Because of this, researchers have been challenged to develop valid and reliable instruments to measure disability and impairment among youth. These research endeavors are essential in understanding how chronic health conditions and disabilities affect youth, their families, and the health care system. One measure that holds considerable potential is the World Health Organization Disability Assessment Schedule 2.0 (WHODAS 2.0) [2,3].

The WHODAS 2.0 was developed from a comprehensive set of items derived on the basis of the International Classification of Functioning, Disability and Health [4] to form a generic, self-report measure that assesses global disability in the previous 30 days [2,3]. Two versions of the WHODAS 2.0 have been developed, which are 36- and 12-items, respectively. The global adoption of the WHODAS 2.0 has resulted in its implementation with respondents 12 to 85 years of age with a variety of disability-related conditions in over 30 languages [3,5,6]. These measures can be completed by respondents, proxy informants, or clinicians; and either by self-administration or via interview. Domains of functioning assessed by the WHODAS 2.0 are: cognition, mobility, self-care, getting along, life activities, and participation; with the 12-item version derived through the examination of items most strongly loading on their respective domain-specific factor in the original 36-item version [7]. The six domains of functioning evaluated by the 12 and 36-item versions correlate strongly with the latent construct of global disability and this higher-order factor structure has been replicated for both the 36- and 12-item WHODAS 2.0 [3,8,9].

Evidence pointing to the validity and reliability of the WHODAS 2.0 measure has largely focused on the 36-item version; with studies reporting adequate internal consistency across self-report and proxy informants (α = 0.84 to 0.98) [3,10–12]. More recent work has begun to explore the psychometric properties of the 12-item WHODAS 2.0. For example, work by Andrews et al. (2009) compared a one factor, first-order solution to that of a single second-order factor solution that included a higher-order disability factor represented by six first-order factors/domains of disability. Although the single first-order model demonstrated adequate fit on some of the measurement indices (TFI = 0.97; CFI = 0.98), the single second-order factor solution demonstrated superiority on all fit metrics (TLI = 0.99; CFI = 1.00; SRMR = 0.07; RMSEA = 0.04) [8]. Additional empirical evidence for a single, second-order factor model of the 12-item WHODAS 2.0 has been provided by Kirchberger et al. (2014). Specifically, the authors the authors completed a follow-up survey with 2,077 adults registered with the German MONICA/KORA Myocardial Infarction Registry to discern the feasibility and psychometric appropriateness of the 12-item WHODAS among patients with a history of myocardial infarction [13]. Rasch analysis revealed that all items demonstrated higher loadings on the general factor of disability (0.67 to 0.95) compared to their domain specific factors and item frequencies were very close to their expected probabilities.

Other work by Sousa et al. (2010) used principal components analysis and confirmatory factor analysis to examine the reliability, unidimensionality, and factor structure of the 12-item version across seven samples of older adults (> 65 years), with samples coming from Cuba, Dominican Republic, Venezuela, Mexico, Peru, China and India [14]. While the internal consistency reliability estimates were strong across all countries (α = 0.90 to 0.97) and data from most of the countries fit best with a single-factor solution, the actual fit estimates for both of the single and bifactor first-order factor solutions were marginal, at best (TLI = 0.49 to 0.90; RMSEA = 0.09 to 0.25) [14]. These findings suggest that for the 12-item version of the WHODAS 2.0, a single, second-order factor solution is likely best suited for assessing global disability experiences and the items included in the 12-item WHODAS 2.0, constitute reliable, observable, and discernable indicators of disability status and functional impairment.

Despite being developed for use in adult populations, the WHODAS 2.0 has been used to measure disability in youth as young as 12 years of age [8,9,15]. A study by Hu et al. (2012) has provided some evidence of the appropriateness for implementing the WHODAS 2.0 among adolescents in China. Specifically, the authors assessed the measurement invariance of the 36-item WHODAS 2.0 –that is, the degree to which the measurement properties of the construct under study are equivalent across the groups of interest—between adolescent in-patients and school controls [16]. After one item relating to sexual activity was removed, multiple-group confirmatory factor analysis revealed that the 35-item WHODAS 2.0 was invariant across school-based and inpatient samples of Chinese adolescents. The problem however, is that the WHODAS 2.0 measure was initially developed to assessed disability in adults and, without formally testing for measurement invariance between youth and adults, researchers cannot be certain that the WHODAS 2.0 is performing as intended in youth populations.

Measurement invariance is a prerequisite for making meaningful comparisons between independent groups [17]. Specifically, measurement invariance determines the extent to which a measure demonstrates construct comparability across the groups of interest, is a prerequisite for confirmation that a latent variable can be measured by the same indicators across groups; and therefore, it is critical for interpreting similarities and differences across groups as true and meaningful. Without evaluating the measurement invariance of the WHODAS 2.0 measure in youth compared to adults, observed scores and the extent to which they reflect the underlying distribution of the population cannot be assumed [17–20]. Our team is not aware of any study that examines the measurement invariance of the 12-item WHODAS 2.0 between youth and adults. Rather, previous invariance work has combined youth and adult respondents to examine the invariance of the 12-item version across cultures [14] and patients with major depressive disorder [21]. In an effort to extend the construct comparability of the WHODAS 2.0 and provide empirical evidence for its meaningful use in youth populations, the objective of this study was to test for measurement invariance of the 12-item WHODAS 2.0 across youth (15 to 17 years) and adults (≥ 18 years) using data from a large representative study from Canada.

From a developmental perspective, this will have important implications for the use of the 12-item WHODAS 2.0 in research and clinical practice. Specifically, adolescence is a developmental period that is characterized by extensive biological and physiological changes where an individual’s perception of their body, their bodily attributes and abilities in relation to others (e.g. siblings and peers) is of heightened importance [22]. This may mean that adolescents’ experience or interpretation of their abilities or disability may or may not differ from those of adults, who tend to be less concerned with the perceptions of others [23]. For these reasons, demonstrating the measurement invariance of the 12-item WHODAS 2.0 between youth and adults would allow the opportunity for clinicians to have confidence in the routine use of the measure as a short disability screener in clinical practice and to use the screener to deliver a personalized medicine approach—irrespective of the age of the patient. Equally compelling is that the demonstration of measurement invariance for the WHODAS 2.0 measure between youth and adults would mean that one can empirically justify the use of the measure to track the trajectory of disability experiences among individuals of varying ages and health conditions, over time.

## Materials and Methods

### Data Source

#### Participants

The 12-item WHODAS 2.0 was included in the 2012 Canadian Community Health Survey-Mental Health (CCHS-MH); a national epidemiological study conducted by Statistics Canada (Record Number 5015) [15]. Using a multistage stratified cluster sampling design, a representative sample of respondents ≥ 15 years of age were included (N = 25,113). Individuals residing in the Canadian territories, Aboriginal settlements, institutions, and full-time members of the Canadian Forces were excluded (representing approximately 3% of the target population). Most interviews (87%) were conducted in respondents’ households by an interviewer using computer-assisted personal interviewing. The household-level response rate was 79.8% and the combined household and person response rate was 68.9% [15]. Access to the 2012 CCHS-MH can be requested through a formal application to Statistics Canada through the Social Sciences and Humanities Research Council (http://www.statcan.gc.ca/eng/rdc/process).

Across the total sample of CCHS-MH participants, 27% (n = 6,837) were missing at least some information on the WHODAS 2.0. Specifically, 50 respondents were missing data on all of the WHODAS 2.0 items and were removed from the analysis. To examine characteristics of non-response among our remaining sample (n = 25,063), missed responses on WHODAS 2.0 items were combined and re-coded as 0 (*complete WHODAS 2*.*0 data*), or 1, (*partially missing WHODAS 2*.*0 data)* and modeled as a dependent variable in a logistic regression analysis. The independent variables entered into the logistic regression model represented a number of respondent characteristics, including: age, sex, immigrant status, marital status, employment status, educational attainment, household income and whether or not the respondent was currently living with a chronic health condition (e.g., arthritis, asthma, diabetes, epilepsy). Missing data were not associated with any of the respondent characteristics considered and therefore, were assumed to be missing at random. Our dataset included 1,265 youth 15 to 17 years of age and 23,798 adults aged ≥ 18 years. To pursue our research objectives and as many aspects of confirmatory factor analyses, our main analysis technique, are related to sample size, we balanced our youth and adult sub-samples by selecting a simple random sample of 1,265 adults using the SURVEYSELECT procedure in SAS 9.4. Thus, our final sample for analysis includes 1,265 youth (15 to 17 years) and 1,265 adults aged ≥ 18 years. Six percent of respondents belonging to the youth sub-sample and 28% of respondents belonging to the adult sub-sample were missing data on at least one of the WHODAS 2.0 items. Our strategy for the treatment of missing data is described in the *Evaluation Criteria*.

#### Instrument

The measure used in the present study is the 12-item version of the WHODAS 2.0; which is a global measure of disability that incorporates the assessment of six domains of functioning. These domains of functioning are: cognition, mobility, self-care, getting along, life activities, and participation. In this regard, the measure consists of a single-second order factor structure, where ‘disability’ represents the single second order factor whose score is given by the respondent’s combined score on the six first-order factors (i.e. domains). Preceded by the statement, “In the past 30 days, how much difficulty did you have in…,”participants were asked to respond to each of the 12 items using a five point scale that ranged from ‘0’ (none) to ‘4’ (extreme/cannot do). Table 1 details the item characteristics of the WHODAS 2.0, as well as the average item scores and their standard deviations across our youth and adult sub-samples. Mean WHODAS 2.0 items scores for youth ranged from 1.00 to 1.19 and from 1.09 to 1.54 for adults, respectively. Median scores for each item for youth and adults was 1.00. Overall disability score can range from 0–100, where 0 is no disability and 100 is complete disability.

**Table 1:** Description of the WHODAS 2.0 Items.

		Youth		Adults	
Domain (Factor)	Item Number and Text	Mean	Standard Deviation	Mean	Standard Deviation
Mobility (F1)	1. Standing for long periods such as 30 minutes?	1.16	0.52	1.46	1.05
	7. Walking a long distance such as a kilometer (or equivalent)?	1.12	0.50	1.54	1.18
Life Activities (F2)	2. Taking care of your household responsibilities?	1.19	0.53	1.33	0.82
	12. Your day to day work?	1.28	0.62	1.29	0.80
Cognition (F3)	3. Learning a new task, for example learning how to get to a new place?	1.14	0.43	1.15	0.56
	6. Concentrating on doing something for 10 minutes?	1.31	0.68	1.19	0.58
Participation (F4)	4. How much of a problem did you have joining in community activities (for example, festivals, religious or other activities) in the same way as anyone else can?	1.15	0.49	1.26	0.77
	5. How much have you been emotionally affected by your health problems?	1.18	0.52	1.39	0.82
Self-care	8. Washing your whole body?	1.00	0.07	1.09	0.41
(F5)	9. Getting dressed?	1.01	0.10	1.09	0.38
Getting Along (F6)	10. Dealing with people you do not know?	1.19	0.54	1.15	0.56
	11. Maintaining a friendship?	1.09	0.36	1.09	0.47

The WHODAS 2.0 is available at http://www.who.int/classifications/icf/whodasii/en/.

Respondents were informed about the objectives of the study, its content focus, privacy, confidentiality, and the voluntary nature of the survey and gave their informed verbal consent to the Statistics Canada personnel to participate. Respondents were additionally informed that participant confidentiality and privacy were guaranteed by Statistics Canada under the *Statistics Act*. The verbal consent was coded by the interviewer on respondents interview documentation/electronic file [24]. Analyses were approved by the Hamilton Integrated Research Ethics Board.

### Analytical Procedure

Multiple-group confirmatory factor analysis incorporating a categorical variable framework [25] was used to examine measurement invariance of the WHODAS 2.0. The analysis approach includes a hierarchical set of increasingly stringent models that implement a sequenced set of equality constraints between youth (15 to 17 years) and adults (≥ 18 years). The following sequential testing and model specification strategy was devised from published guidelines for establishing measurement invariance of higher-order factor models comprised of categorical items [25,26]: (i) configural invariance (model 1) imposes no equality constraints on parameters [25] and was used as the origin for more complex models to be tested [27]; (ii) weak invariance (i.e., constrained factor loadings) examines the extent to which the magnitude of the factor loadings (Λ) for particular items (model 2) and first-order factors (model 3) are the same between groups [17] and is a prerequisite for making valid comparisons [28]; (iii) strong invariance (i.e., constrained item thresholds/intercepts) tests for evidence that item thresholds (ν) and first-order factor intercepts (τ) are invariant between groups (model 4) [17] and verifies whether mean differences at the item-level are fully explained by mean differences at the higher-order factor-level; and, (iv) strict invariance (i.e., constrained residual and factor variances) is performed to determine whether the variances (θ) of the regression equations for each item (model 5) and first-order factors (model 6) are equivalent across groups. Strict equivalence between groups is required for defensible item-score comparisons (i.e., average item scores) between groups [19].

### Evaluation Criterion

Measurement invariance was considered to be present when, after imposing a parameter constraint, there was no appreciable worsening of model fit. If this condition was met, invariance testing proceeded to the application of the next equality constraint. If there was significant worsening of fit, modification indices were reviewed. If the output suggested that removing constraints on non-invariant parameters would improve model fit, these constraints were removed and allowed to vary freely. This re-specified model was then tested against the less constrained model to determine measurement invariance of the freely estimate parameter.

Due to the ordered categorical nature of responses for the WHODAS 2.0, the confirmatory factor model was estimated with a weighted least squares means and variance adjusted estimator. This estimator uses a diagonal weight matrix and pairwise deletion to account for missing data and generate robust parameter estimates [29,30]. Model fit was based on the following indices: Comparative Fit Index (CFI), Tucker-Lewis Index (TLI), and Root Mean Square Error of Approximation (RMSEA) and 90% confidence interval [31–33]. Thresholds for model fit were defined using the following cutoffs: CFI and TLI > 0.95, and RMSEA < 0.06 [27,30,33,34] Adequate model fit was achieved if at least two of these three indices met their respective cutoff points [35–37]. Presence of invariance at each level of analysis was determined through the estimation and evaluation of multiple change tests, including the χ^2^ difference test, change in the CFI, TLI, and RMSEA. Research has shown that unlike the χ^2^ difference test, the change in the CFI, TLI and RMSEA are not influenced by sample size [31]. Given this information and based on previous literature, measurement invariance was considered established when two or more of following were satisfied: the χ^2^ difference test resulted in a p-value > .05; ΔCFI < -0.010; ΔTLI < -0.010; ΔRMSEA = 0.015 [31,35–38]. Thus, measurement invariance was determined based on statistical and practical significance [31].

Descriptive statistics and comparisons between youth and adult samples were calculated using SAS 9.4 (SAS Institute Inc., United States). Analyses associated with measurement invariance testing were performed with M*plus* 7.11 (Muthén & Muthén, United States).

## Results

### Sample Characteristics

As shown in Table 2, the mean age of youth was 16.0 (*SE* 0.1) years and for adults it was 47.1 (0.2) years. Almost half of the overall sample (49.3%) was male; a characteristic which was maintained in the youth and adult sub-samples. The average household income did not significantly differ across our youth and adult sub-samples. However, as expected, the adults were more likely to have a chronic health condition, be married or in a common-law relationship, graduated from secondary school, employed full-time, and to identify as an immigrant.

**Table 2:** Characteristics of Participants in the Canadian Community Health Survey-Mental Health.

Characteristic	Youth	Adult	Adult
	(*n* = 1,265)	(full sample, *n* = 23,798)	(invariance sample, *n* = 1,265)
Age, years	16.0 (0.1)	47.1 (0.2)*	46.7 (0.8)*
Male, %	51.9	49.1	50.9
Chronic Health Condition, %	40.9	60.0*	58.9*
Married^a^, %	0.3	63.1*	65.1*
Secondary Graduate, %	18.0	85.2*	84.7*
Full-time Employment, %	11.7	84.7*	87.2*
Immigrant, %	10.3	25.9*	24.6*
Household Income^b^, CAD	82,570 (2418.8)	80,153 (1091.1)	78,663 (3330.8)
WHODAS 2.0	4.5 (0.3)	5.4 (0.1)*	4.9 (0.4)

Values are mean (standard error) unless stated otherwise. *P*-Values are for testing whether the sample characteristic is significantly different among adults compared to youth.

^a^ Includes common-law relationships.

^b^ Reported in Canadian dollars per year (CAD).

* *p* < .001

With respect to the average total WHODAS 2.0 scores, results suggest that our youth and adult sub-samples were experiencing minimal disability at the time of data collection. However, total WHODAS 2.0 scores were significantly higher among the adult group, compared to youth.

### Measurement Properties

Table 3 details the polychoric correlational structure of the WHODAS 2.0; demonstrating that each of the respective items was significantly correlated with one another in our youth and adult sub-samples. In addition, the ordinal coefficient alpha for the WHODAS 2.0 items and their 95% confidence intervals among our youth and adult sub-samples were 0.92 (0.91, 0.93) and 0.95 (0.94, 0.96), respectively. [39,40] These estimates confirm an appropriate level of unidimensionality at the second-order-factor level of the WHODAS 2.0.

**Table 3:** WHODAS 2.0 Correlation Matrix.

	Q1	Q2	Q3	Q4	Q5	Q6	Q7	Q8	Q9	Q10	Q11	Q12
**Q1**		0.73	0.44	0.55	0.58	0.36	0.82	0.82	0.81	0.40	0.33	0.59
**Q2**	0.52		0.53	0.73	0.63	0.49	0.72	0.76	0.70	0.48	0.62	0.79
**Q3**	0.41	0.56		0.61	0.44	0.57	0.48	0.50	0.49	0.58	0.56	0.61
**Q4**	0.48	0.46	0.50		0.65	0.56	0.64	0.70	0.68	0.50	0.79	0.75
**Q5**	0.40	0.34	0.34	0.55		0.66	0.49	0.62	0.59	0.50	0.58	0.68
**Q6**	0.35	0.53	0.50	0.47	0.47		0.40	0.44	0.45	0.56	0.55	0.72
**Q7**	0.72	0.45	0.46	0.60	0.44	0.34		0.81	0.79	0.31	0.38	0.65
**Q8**	0.63	0.66	0.35	0.56	0.34	0.37	0.68		0.95	0.42	0.52	0.64
**Q9**	0.66	0.56	0.30	0.47	0.51	0.17	0.71	0.90		0.44	0.54	0.65
**Q10**	0.37	0.35	0.54	0.61	0.51	0.56	0.34	0.36	0.28		0.71	0.53
**Q11**	0.41	0.41	0.58	0.63	0.40	0.44	0.45	0.51	0.44	0.56		0.65
**Q12**	0.37	0.53	0.50	0.51	0.51	0.68	0.42	0.29	0.57	0.56	0.55	

Note: Top diagonal = adults, bottom diagonal = youth. Table represents the polychoric correlations among WHODAS 12.0 items between adults and youth. All correlations are significant at p < .001.

### Measurement Invariance

To confirm the appropriateness of estimating the measurement invariance of the 12-item, second-order factor structure, we evaluated the fit of previously published, higher-order factor, single first-order factor, and six first-order factor models across our youth and adult sub-samples. As expected, the higher-order factor model fit the youth (χ^2^(48) = 95.55; CFI = 0.979; TLI = 0.971; RMSEA = 0.028 [0.020, 0.036]) and adult (χ^2^(48) = 138.13; CFI = 0.984; TLI = 0.979; RMSEA = 0.039 [0.031, 0.046]) sub-samples best, followed by the single first-order factor model (youth: χ^2^(54) = 126.00; CFI = 0.968; TLI = 0.960; RMSEA = 0.032 [0.025, 0.040]; adults: χ^2^(54) = 238.44; CFI = 0.968; TLI = 0.961; RMSEA = 0.052 [0.045, 0.059]). The six-first-order factor model revealed a covariance matrix that was not positive definite for the youth (χ^2^(39) = 58.04; CFI = 0.991; TLI = 0.985; RMSEA = 0.020 [0.007, 0.030]) and adult (χ^2^(39) = 73.62; CFI = 0.994; TLI = 0.990; RMSEA = 0.027 [0.017, 0.036]) sub-samples; providing evidence for the appropriateness of a higher-order factor structure for the 12-item WHODAS 2.0 among adults and youth.

We proceeded to test the measurement invariance of the 12-item WHODAS following the recommendations by the measurement invariance literature. Specifically, we began with the fitting of independent models for our youth and adult samples, which served as the baseline from which measurement invariance was estimated and confirmed. These baseline models imposed no equality constraints on any parameters of interest and model identification is achieved by setting one item loading on each first-order factor to 1 and one first-order factor loading on the second-order factor to 1. Results of these baseline models can be found in Table 4. Model fit indices suggested adequate fit among these models for adults and youth; suggesting that the overall factor structure of the WHODAS 2.0 is appropriate for our sub-samples of interest. Results of our increasingly restrictive measurement invariance models follow the baseline model information in Table 4.

**Table 4:** WHODAS 2.0 Baseline Model Fit Results and Tests of Measurement Invariance.

Model	χ^2^ (df) P-value	CFI	TLI	RMSEA(90% CI)	Δχ^2^ (df) P-value	ΔCFI	ΔTLI	ΔRMSEA
Independent Baseline Models								
Youth	95.55 (48) < .001	0.979	0.971	0.028 (0.020, 0.036)	-	-	-	-
Adults	138.13 (48) < .001	0.984	0.979	0.039 (0.031, 0.046)	-	-	-	-
**Measurement Invariance Models**								
*Configural Invariance*								
Model 1	523.60 (122) < .001	0.967	0.964	0.051 (0.047, 0.056)	-	-	-	-
*Weak Invariance (Constrained Factor Loadings for Items and First-Order Factors)*								
Model 2	508.78 (128) < .001	0.969	0.968	0.049 (0.044, 0.053)	15.22 (6) .019	0.002	0.004	-0.002
Model 3	502.50 (132) < .001	0.969	0.969	0.047 (0.043, 0.052)	3.93 (4) .416	0.000	0.001	-0.002
*Strong Invariance (Constrained Item Thresholds and First-Order Factor Intercepts)*								
Model 4	640 (138) < .001	0.959	0.960	0.054 (0.049, 0.058)	101.28 (6) < .001	-0.010	-0.009	0.007
*Strict Invariance (Constrained Residual and Factor Variances)*								
Model 5	720.49(150) < .001	0.953	0.959	0.055(0.051, 0.059)	98.50 (12) < .001	-0.006	-0.001	0.001
Model 6	769.04 (159) < .001	0.950	0.958	0.055 (0.051, 0.059)	81.24 (9) < .001	-0.003	-0.001	0.000

CI = confidence interval

**Fig 1:**
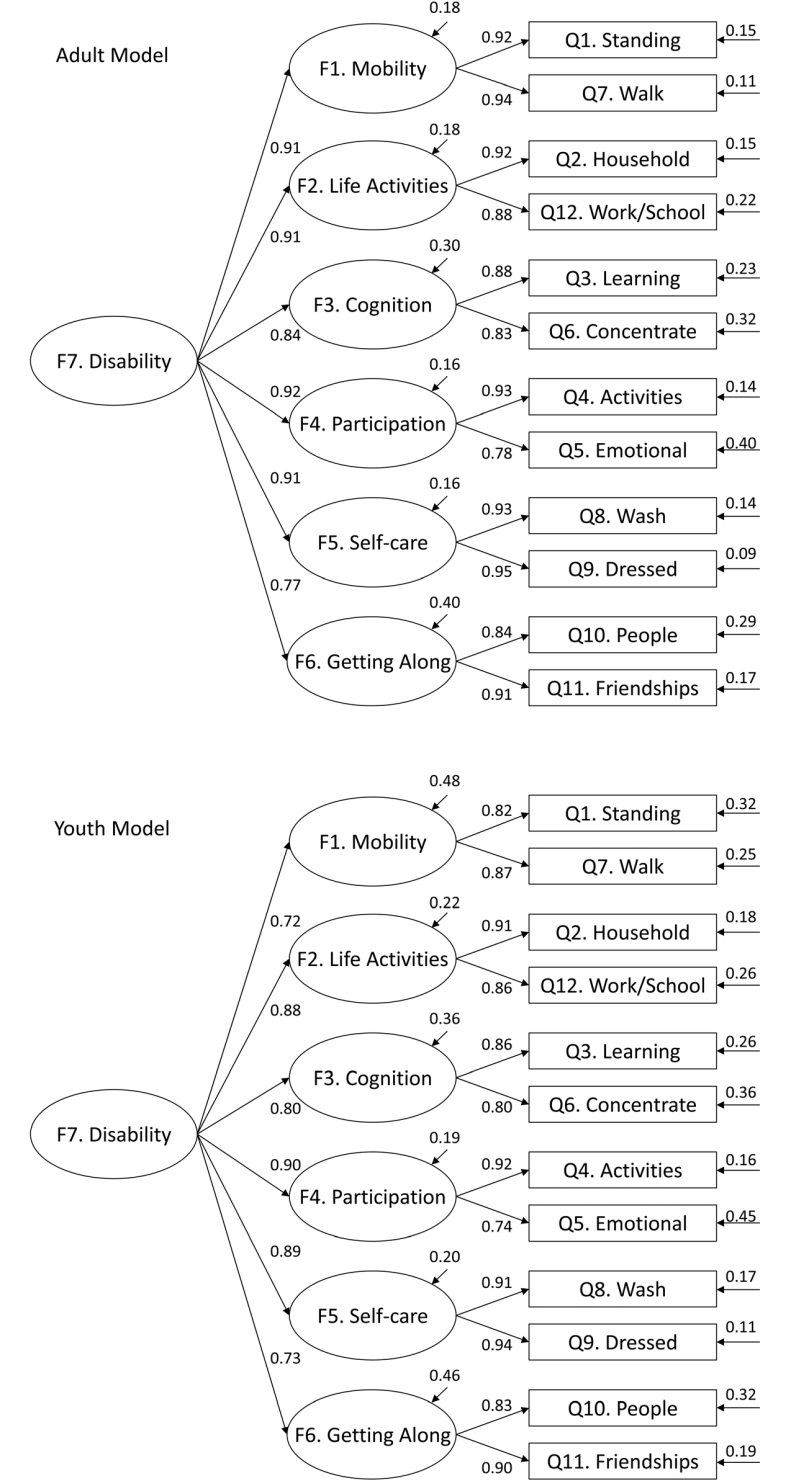
Results of the Invariant Second-Order Factor Model of the WHODAS 2.0. This figure shows the standardized estimates of the higher-order factor structure among adults and youth following the estimation of measurement invariance.

Model 1 estimated and demonstrated configural invariance of the WHODAS 2.0 between youth and adults by freely estimating all parameters (except those constrained for model identification) simultaneously in both groups. Estimates indicated adequate model fit: χ^2^(122) = 523.60, p < .001; CFI = 0.967; TLI = 0.964; RMSEA = 0.051 [0.047, 0.056]. This suggests that the same number and pattern of factors or constructs is present in both the youth and adult sub-samples. Models 2 and 3 estimated the extent to which the 12-item WHODAS 2.0 measure meets weak invariance criteria; and more specifically, whether the first and second-order factor loadings had the same meaning across our groups and whether the items used to estimate these factor loadings have the same meaning across groups. Constraining factor loadings for the indicator items and the first-order factors meant that weak invariance was, in fact, established at the first (**Δ**CFI = 0.002; **Δ**TLI = 0.004; **Δ**RMSEA = -0.002) and second-order factor level (**Δ**CFI = 0.000; **Δ**TLI = 0.001; **Δ**RMSEA = -0.002). Constrained item thresholds and first-order factor intercepts for Model 4 resulted in the demonstration of strong invariance (**Δ**CFI = -0.010; **Δ**TLI = -0.009; **Δ**RMSEA = 0.007), meaning that that item thresholds (ν) and first-order factor intercepts (τ) are invariant between groups. Strict invariance (Model 5) at the item level was evaluated and achieved by placing constraints on the residual variances of the indicator items (**Δ**CFI = -0.006; **Δ**TLI = -0.001; **Δ**RMSEA = 0.001). Finally, strict invariance at the higher-order factor level (Model 6) was achieved when constraining the first-order factor variances (**Δ**CFI = -0.003; **Δ** TLI = -0.001; **Δ**RMSEA = 0.000). Strict invariance findings suggest that item-score comparisons (i.e., average item scores) can be made between youth and adults. Standardized parameter estimates for the final youth and adult WHODAS 2.0 models are illustrated in Fig 1.

To further confirm the appropriateness of the higher-order factor structure among our adult and youth sub-samples, we estimated the substantive influence of the first and second order factors on the WHODAS 2.0 items using the Schmid-Leiman transformation. [41] Results of this transformation can be found in Table 5. Specifically, the table details the how much variance in each of the WHODAS 2.0 items is explained by its first-order (Residualized Loading R^2^) and second-order factor (Higher-Order R^2^) [41,42]. For example, among adults, approximately 70% of the variation in item Q2 (“Taking care of your household responsibilities”) is explained by the second-order factor ‘Disability’. Similarly, 18% of the variation in this item among adults is explained by the first-order factor, ‘Life Activities’. Generally speaking, a greater proportion of the variance in the WHODAS 2.0 items is accounted for by the second-order factor in both the youth and adult samples. However, the percent variation accounted for by the second-order factor does differ across the WHODAS domains, suggesting that retaining a second order-factor structure for our measurement invariance testing, is appropriate.

**Table 5:** Schmid-Leiman Transformation of WHODAS 2.0 items.

		Youth		Adults	
Domain (Factor)	Item Number	Residualized Loading R^2^	Higher-Order R^2^	Residualized Loading R^2^	Higher-Order R^2^
Mobility (F1)	Q1	0.32	0.35	0.15	0.70
	Q7	0.36	0.39	0.16	0.73
Life Activities (F2)	Q2	0.18	0.64	0.15	0.70
	Q12	0.16	0.57	0.14	0.64
Cognition (F3)	Q3	0.27	0.47	0.23	0.55
	Q6	0.23	0.41	0.21	0.49
Participation (F4)	Q4	0.16	0.69	0.14	0.73
	Q5	0.10	0.44	0.10	0.51
Self-care (F5)	Q8	0.17	0.66	0.14	0.72
	Q9	0.18	0.70	0.14	0.75
Getting Along (F6)	Q10	0.32	0.37	0.28	0.42
	Q11	0.37	0.43	0.33	0.49

## Discussion

Using contemporary data from a representative survey in Canada, the higher-order factor structure of the WHODAS 2.0 was confirmed in a nationally representative sample of youth aged 15 to 17 years and adults aged ≥ 18 years. The confirmation of this higher-order structure replicated the findings from other epidemiological studies [3,8]. Moreover, findings provided evidence to suggest that the WHODAS 2.0 demonstrated configural, weak, strong and strict invariance between youth and adults, indicating that the WHODAS 2.0 items were perceived similarly between youth and adults and that the second-order factor structure is appropriate for both samples.

Researchers have argued that *strict* measurement invariance is not required for substantive analyses if at least a subset of parameters is determined to be invariant (i.e., *partial* invariance). Indeed, previous research has demonstrated that the presence of two invariant parameters—two weak (equal factor loadings) and strong (equal thresholds/intercepts)–is sufficient for meaningful comparisons between groups [43]. The finding of strict measurement invariance in the present study has met and exceeded these psychometric requirements. These findings have practical implications for clinicians and researchers interested using the WHODAS 2.0 to assess disability in youth. Specifically, given that measurement invariance was established, observed differences in mean disability scores can be attributed to real differences in individual ratings. When disability among youth is evaluated, individual global disability scores can be compared meaningfully across subgroups of youth and with population norms. In addition, the empirical evidence from this study as well as those documenting its sensitivity for detecting change in disability over time, suggests that the 12-item WHODAS 2.0 has practical utility for following the trajectory of disability experiences from youth into adulthood for both clinical and representative, population-based samples.

This study is strengthened by its broad population coverage which included a large, representative sample of participants. However, it is noteworthy that despite the contribution to establishing the applicability of the WHODAS 2.0 among youth, data from the CCHS-MH were limited to youth aged 15 to 17 years of age. Thus, it is not possible to extrapolate these findings to younger children. Additional invariance testing is needed to support the use of the WHODAS 2.0 in children, as well as children and youth of both genders. In addition, it is important to note that the testing of measurement invariance is required for defensible comparisons to be made on a measure of interest across groups. However, in this regard, it is only able to consider the invariance of a measure on a singular observed characteristic of interest (e.g., youth vs. adults); and therefore, does not consider any other potential unobserved population heterogeneity that may account for differential item responses. Now that measurement invariance of the WHODAS 2.0 measure has been established across youth and adults, future work should incorporate advanced mixture modeling techniques suggested by Brown [42] and Lubke and Muthén [44] to determine the extent to which unobserved population heterogeneity may be contributing to the invariance results demonstrated in the present study. Finally, confirmatory factor analysis of alternate models—including a single, first-order factor model and a six, first-order factor model—as well as the results from the Schmid-Leiman transformation suggest that a significant proportion of the variation in WHODAS items are accounted for by the higher order disability factor; with the six, single-order factor model producing a non-positive definite correlation/covariance matrix [41]. From a methodological and clinical perspective, this suggests that researchers and clinicians should err on the side of caution when interpreting single order factor scores (i.e., subscale scores) as singular metrics of disability.

## Conclusion

Findings from the current study extend previous research reporting the reliability and validity of the 12-item WHODAS 2.0 as a measure of global disability in epidemiological studies to include youth as young as 15 years of age. Additional research examining the feasibility, reliability, and validity of the WHODAS 2.0 in younger children is warranted and would make an important contribution to measuring disability in child populations.
